# Evaluating the quality of informed consent and contemporary clinical practices by medical doctors in South Africa: An empirical study

**DOI:** 10.1186/1472-6939-14-S1-S3

**Published:** 2013-12-19

**Authors:** Sylvester C Chima

**Affiliations:** 1 Programme of Bio & Research Ethics and Medical Law, Nelson R Mandela School of Medicine and School of Nursing and Public Health College of Health Sciences, University of KwaZulu Natal Durban, South Africa

**Keywords:** Africa, Autonomy, Patients' rights, Informed consent, Doctors, Empirical ethics, Nurses, Laws, Regulations

## Abstract

**Background:**

Informed consent is a legal and ethical doctrine derived from the principle of respect for autonomy. Generally two rights derived from autonomy are accorded legal protection. The constitutional right to bodily integrity followed by the right to bodily well-being, protected by professional negligence rules. Therefore healthcare professionals treating patients' without valid consent may be guilty of infringing patients' rights. Many challenges are experienced by doctors obtaining informed consent in complex multicultural societies like South Africa. These include different cultural ethos, multilingualism, poverty, education, unfamiliarity with libertarian rights based autonomy, and power asymmetry between doctors and patients. All of which could impact on the ability of doctors to obtain legally valid informed consent.

**Methods:**

The objective of this study was to evaluate whether the quality of informed consent obtained by doctors practicing in South Africa is consistent with international ethical standards and local regulations. Responses from 946 participants including doctors, nurses and patients was analyzed, using a semi-structured self-administered questionnaire and person triangulation in selected public hospitals in Durban, KwaZulu-Natal, South Africa.

**Results:**

The median age of 168 doctors participating was 30 years with 51% females, 28% interns, 16% medical officers, 26% registrars, 30% consultant/specialists. A broad range of clinical specialties were represented. Challenges to informed consent practice include language difficulties, lack of interpreters, workload, and time constraints. Doctors spent 5-10 minutes on consent, disclosed most information required to patients, however knowledge of essential local laws was inadequate. Informed consent aggregate scores (ICAS) showed that interns/registrars scored lower than consultants/specialists. ICAS scores were statistically significant by specialty (p = 0.005), with radiologists and anaesthetists scoring lowest, while internists, GPs and obstetricians/gynaecologists scored highest. Comparative ICAS scores showed that professional nurses scored significantly lower than doctors (p ≤ 0.001).

**Conclusions:**

This study shows that though doctors had general knowledge of informed consent requirements, execution in practice was inadequate, with deficiency in knowledge of basic local laws and regulations. Remedying identified deficiencies may require a 'corps' of interpreters in local hospitals to assist doctors in dealing with language difficulties, and continuing education in medical law and ethics to improve informed consent practices and overall quality of healthcare service delivery.

## Background

Informed consent is a legal doctrine in medical practice, derived from the ethical principle of respect for autonomy. It has been argued that "prima facie, every competent adult has the right to decide whether to consent or refuse any medical treatment, even if such refusal could lead to death" [1]. However, this right to respect for autonomy is a rebuttable right, which could be overridden under certain conditions such as where there is temporary or permanent mental incapacity due to unconsciousness, infancy, or severe mental retardation [2]. Respect for autonomy in medical law and ethics refers to self-determination or freedom of choice. This ethical principle that each person has a right to determine what can be done to his or her own body during medical treatment has found expression in many national health statutes and international ethical codes through the doctrine of informed consent. Autonomy itself has never been found to be a legally enforceable right; rather two other rights derived from the principle of respect for autonomy have been universally accorded legal protection. The first is the right to bodily integrity protected by legal rules against assault or battery. The second is the right to bodily well-being, protected by professional negligence rules [2,3]. Next to these is the right to liberty or the condition of being free [4]. A patient's right to autonomy and informed consent during medical treatment was popularized as a legal doctrine by Cardozo J in the *Schloendorf *case [5] where he opined that, "every human being of adult years and sound mind has a right to determine what shall be done with his own body, and the surgeon who performs and operation without his patients consent commits and assault for which he is liable in damages". This opinion was later reaffirmed by the US Supreme Court in the *Cruzan *case [6] where the court stated that:

N*o right is held more sacred or is more carefully guarded by the common law, than the right of every individual to the possession and control of his own person, free from all restraint or interference of another*.

Therefore a physician, who treats a patient without consent or exceeds the consent given by a patient, may be guilty of infringing the patient's right to bodily integrity and bodily well being [7]. As summarized by Lord Goff in *Airedale NHS Trust v. Bland *[1]:

*The first point to make is that it is unlawful so as to constitute both the tort and crime of battery, to administer medical treatment to an adult who is conscious and of sound mind, without his consent...such a patient is completely at liberty to decline to undergo treatment, even if the result of his doing so will be that he will die*.

## Informed consent as an ethical doctrine

The UNESCO International Bioethics Committee (IBC) report on consent argues that, 'autonomy implies responsibility'. That the power to decide for one's self entails *ipso facto *acceptance of the consequences of one's actions, which can have far reaching consequences especially in matters of health [8]. Therefore, a person needs to be informed of the precise consequences of his/her choice, and this in turn leads one to consider the conditions under which consent is obtained. Respect for the autonomy of persons making decisions, while taking responsibility for those decisions, is closely aligned to article 1 of the Universal Declaration of Human Rights (UDHR) which holds that all human beings are born free and equal in dignity and rights. They are endowed with reason and conscience and should act towards one another in a spirit of brotherhood [8,9]. In view of the foregoing, it could be argued that the doctrine of informed consent has evolved into a rule of law that requires that no diagnostic or therapeutic procedure should be performed on a patient, without full disclosure of the risks of the procedure and any alternatives to it, prior to giving consent.

## The nature of informed consent

Informed consent has been defined as an autonomous authorisation by individuals of a medical intervention [10,11]. Others have described a complementary view of informed consent as a conversation that follows specific rules [12]. Such a conversation, should ideally be initiated by the physician or healthcare professional and involves transparency, engagement by both parties, and continues throughout the period of healthcare intervention. This conversation may also require evidence that it occurred in the form of a witnessed signature, co-signed consent documents, or medical progress notes [12]. As a general rule, medical treatment should not proceed unless the doctor has first obtained the patient's consent which may be either express or implied. The consent given by a patient can be withdrawn at anytime [13] and could be vitiated by any change in circumstances, which are not communicated to and approved by the individual consenting.

## What makes consent valid?

Generally, for consent to be considered valid or truly informed, five key requirements must be fulfilled [10,11,14]. These would be:

(a) *Information disclosure: *provision of adequate information

(b) *Competence: *capacity to understand that information

(c) *Voluntariness: *decision making in the absence of coercion or deception

(d) *Comprehension: *understanding of information provided

(e) *Consent: *agreement to the proposed treatment or procedure

It has been argued that informing the patient must not be simply a ritual recitation of the contents of a written document. Rather the healthcare professional must try to convey the information, whether orally or in writing, in language that suits the individual's level of understanding [15]. The healthcare professional obtaining consent should bear in mind that the prospective subject's ability to understand the information necessary to give consent depends on that individual's maturity, intelligence, educational level, and belief system. It also depends on the clinician's ability and willingness to communicate with patience and sensitivity [16]. According to the US District Court of Appeal in *Canterbury v. Spence *[17]:

*The patient's right to self-determination can be effectively exercised only if the patient possesses enough information to enable intelligent choice...True consent to what happens to one's self is the informed exercise of choice and that entails an opportunity to evaluate knowledgeably the options available and the risks attendant upon each. From these axiomatic considerations springs the need, and in turn the requirement, of a reasonable divulgence by the physician to the patient to make such a decision possible*.

It was further asserted in the case of *Salgo v. Leland Stanford University *that: "A physician may violate his duty to his patient and subject himself to liability if he withholds any facts which are necessary to form the basis of an intelligent consent by the patient to proposed treatment" [18]. Because of this potential for violation of patient's rights and dignity during the informed consent process, it has been suggested the quality of informed consent given by patients during various clinical encounters, should be scientifically investigated for validity, completeness, and consistency with established ethical and legal principles. [19,20].

## Informed consent in South Africa

Informed consent before medical procedures is constitutionally protected right in South Africa. This was demonstrated in the case of *Minister of Safety and Security v. Xaba *[21]. Here the police wanted a court order to compel an accused person to undergo a surgical procedure in order to obtain a bullet to be used in evidence against the accused. The Court refused this request; arguing that such and order would violate the defendant's constitutional rights to bodily and psychological integrity, including the right to security and control of one's body [22]. Patients consent, as a requirement for all lawful medical interventions, is a well-established principle in South African common law [23]. The earliest cases in this area were *Stoffberg v. Elliot *1923 [24] and *Esterhuizen v. Administrator Transvaal *1957 [25]. In the former case a patient whose member was wrongfully amputated due to penile cancer without informed consent, sued his doctors for damages in action for assault. While instructing the jury, Watermeyer J opined that:

*In the eyes of the law, every person has certain absolute rights, which the law protects. They are not dependent upon a statute or upon a contract, but they are rights to be respected, and one of those rights is the right of absolute security of the person....Any bodily interference with or restraint of a man's person which is not justified in law or excused by law, or consented to, is a wrong, and for that wrong the person whose body has been interfered with has a right to claim such damages as he can prove he has suffered owing to that interference*.

In the case of *Esterhuizen v. Administrator Transvaal*, a 10-year-old child diagnosed with Kaposi's sarcoma was initially treated with superficial radiation with her parents' consent. However, following recurrence of the tumour she was subjected to radical radiation therapy which resulted in severe burns necessitating amputation of her limbs. The Court held that while the superficial radiation was duly performed with appropriate consent from the parents, the latter procedure was performed without the informed consent of the child's guardians. The court rejected the defence arguments for implied consent based on the fact that her parents had previously consented to a similar treatment, as well as arguments that the treatment was in the child's best interest. Holding that because the radical treatment was vastly different from the prior superficial radiation, it was necessary that the child's parent should have been adequately informed of the dangers inherent in the new treatment, before such consent to be considered valid [25]. A more recent judgment in the case of *Castell v. DeGreef *[26] by Ackerman J seems to have consolidated the doctrine of informed consent into South African jurisprudence. The consequences of the latter decision on South African medical law were that the following principles have generally been adopted into the clinical practice of medicine locally [22,27]:

- a shift from medical paternalism to patient autonomy

- a shift from the 'reasonable doctor' standard to the 'prudent patient' standard

- a shift in disclosure to the 'material risk' standard, where the level of disclosure required is what a reasonable patient would consider pertinent before making a decision

It has been suggested that the Court appears to place the patients' informed consent within the framework of *volenti non fit injuria *or voluntary assumption of risk rather than delict [22,27]. The *National Health Act *(NHA) promulgated in 2003 [28] codified the requirements for informed consent into South African law. Section 7 of this act stipulates that health services may not be provided to a healthcare user without the user's informed consent, unless "the user is unable to give informed consent and such consent is given by another person, mandated by the user in writing to grant consent on his or her behalf; or authorized to give such consent in terms of any law or court order; or where the user is unable to give informed consent and no person is mandated or authorized to give such consent" [22,28]. The law further requires that every health care provider must inform a user of "the user's health status except in circumstances where there is substantial evidence that the disclosure of the user's health status would be contrary to the best interests of the user" [28]. Section 6 of the NHA stipulates that information disclosed to patients must include the following:

(a) The range of diagnostic procedures and treatment options generally available to the user.

(b) The benefits, risks, and consequences generally associated with each option; and

(c) The user's right to refuse health services and explain the implications, risks, obligations of such refusal [22,28]. The NHA also requires that the health care providers must inform the user of this information in a language that the user understands and in a manner which takes into account the user's level of literacy [28].

## The potential impact of the socio-cultural milieu in South Africa on informed consent

In South Africa about 25% of the population is unemployed, with a low labour force participation rate of 54% compared to a global average of 69% [29]. There are also historical inequities within population groups because of the legacy of apartheid [30,31]. Therefore basic health care is unaffordable or out reach for most of the local population, therefore the majority still patronize traditional healers for healthcare services. It has been suggested that in this environment, the practice of informed consent is light years away for the majority of the black population [30]. Under such circumstances, any offer of medical assistance is often seen as better than nothing, thus encouraging undue influence, coercion and medical paternalism [30,32]. There is a further dichotomy in the organization of the healthcare services in South Africa, which is dual in nature consisting of private hospitals or medical practice patronized by about 20% of the population who can afford health insurance or possess the financial means to pay for private healthcare. Meanwhile the public health services are patronized by the remaining 80% of indigent citizens [33]. This evident dichotomy in healthcare service delivery may also influence the practice of informed consent in South Africa. Furthermore, most African societies being culturally complex and paternalistic in nature may require that approval be obtained from community elders, extended family members, or men/husbands as heads of households, before the actual patients can provide consent [34]. One of the challenges in this environment is how to ensure that informed consent is truly voluntary and that community or surrogate consent is not substituted for individuals' consent [35]. The issues and considerations outlined above present challenges to ensuring that consent provided in clinical practice in African communities is informed, comprehensible and autonomous. For the purposes of this study I have focussed my investigation on evaluating the quality of informed consent practices by medical doctors in public hospitals in South Africa, while taking into consideration the various key elements of informed consent, such as information disclosure, competence, voluntariness and comprehension by patients.

## The benefits of using empirical methods to study informed consent

Sulmasy and Sugarman have described two potential reasons for studying the actual conduct of a group with regards to compliance with moral and ethical dilemmas. The first is to establish compliance with existing moral norms, and the second is to determine whether policies and procedures designed to operationalise certain moral norms have been successful [36]. In many countries including South Africa, current law requires that doctors must obtain informed consent from patients before involving them in medical treatment, where informed consent is defined as, "consent for the provision of a specified health service given by a person with legal capacity to do so and who has been informed as contemplated in section 6", NHA [22,28]. However, empirical studies have shown that people generally have problems in understanding the risks and benefits of medical treatment and decision making, and this could impact on the actual application of existing laws [37]. For example, a previous study on Dutch nurses charged with taking care of nursing home residents with due consideration to patients rights and respect for autonomy, revealed that these nurses did not comply with existing regulations [38]. Based on such observations, it has been suggested that to guide action; ethical guidelines must be based in reality and should be formulated in such a way that they are continuous with accepted moral norms [39]. Further, it is has been suggested that empirical ethics should be used to defend or criticize concrete moral principles or practices rather than make general claims about moral concepts [40]. Consequently, in recent times, applied ethicists have shifted towards combining empirical, especially social scientific research methods with normative ethical analysis. Proponents of this approach to empirical ethics have argued that the study of people's actual moral beliefs, behaviour and reasoning should be the starting point of ethics. It is acknowledged that the methodologies of the social sciences, especially quantitative and qualitative research, using surveys, interviews and questionnaires is probably the best way to map the reality of peoples actual moral norms [41]. In view of the above I have used the methodology of empirical ethics, by means of a quantitative semi-structured questionnaire based survey and person triangulation [42,43] to study the contemporary practice of informed consent amongst healthcare professionals and patients in KwaZulu-Natal (KZN) province, South Africa. Here I report the result of findings from medical doctors practising in Ethekwini metropolitan municipality (Durban), KZN.

## Methods

### Objectives of the study

The general objective of this study was to evaluate the quality of informed consent obtained by doctors and nurses from patients attending public hospitals in South Africa. Specifically I wanted to establish whether sufficient information was provided to patients before consent is sought. To establish whether patients involved in clinical procedures understand the information given to them. To establish whether consent is obtained from patients is voluntary, and to confirm if the informed consent provided by patients attending public hospitals in South Africa is truly valid.

### Research design

This study was a descriptive cross-sectional study in contemporary clinical practice settings. I also tried to apply the technique of triangulation [42,43] by obtaining data from medical doctors, professional nurses, and patients simultaneously using separate semi-structured questionnaires. Questionnaires were distributed to participants in hospital clinics and wards in real-time during clinic hours. The real-time approach within the hospital environment allowed doctors, nurses and patients to describe their experience with the informed consent process as it is, thereby bringing out the required information. Three trained research assistants distributed and collected the questionnaires from healthcare professionals over a 3-month period from April to June 2012. They also conducted patient interviews using the appropriate questionnaire. To increase the response rate repeated visits was sometimes necessary to collect completed questionnaires from doctors and nurses.

### Research instruments

Data was collected using a self-administered semi-structured questionnaires for healthcare professionals (doctors and nurses), and face-to-face interviews for patients. Two different semi-structured questionnaires were applied to patients and healthcare professionals respectively. The questionnaire for healthcare professionals consisted of 4 sections. The first section collected information on participant demographics. The second section was used to gather information on informed consent practices, such as time spent on obtaining informed consent, patient workload, information disclosed to patients, language and methods used, understanding of information by patients, and challenges faced by healthcare professionals when obtaining informed consent. The third section dealt with generic questions on local laws and regulations on informed consent such as the legal age of consent and standards of information disclosure. The fourth section dealt with understanding and use of implied and presumed consent by doctors and nurses (Additional file 1). The questionnaire for healthcare professionals was informally evaluated by selected healthcare personnel and modified prior to distribution to participants. Questionnaires were distributed by hand to all participants. The study design and research instruments were evaluated and approved by a qualified biostatistician.

### Study location and sampling procedure

The study was conducted in the outpatient clinics and wards at randomly selected public hospitals within Ethekwini metropolitan municipality, KZN. Ethekwini comprises a major urban city (Durban) surrounded by semi-urban areas (townships). The population of this area is approximately 3.2 million (2010 estimate) [44]. According to information from KZN department of Health, there are 17 public hospitals within this municipality ranging from tertiary to district hospitals [45]. Multi-stage stratified random sampling was used to select participating hospitals. The 17 hospitals identified were then arranged alphabetically for stratified sampling. It has been statistically estimated 30% of any population is adequate when conducting a descriptive study [46]. Purposive sampling was also used to include the two central tertiary hospitals within the municipality because they contain the largest number of medical doctors including specialists as well as professional nurses. The rest of the public hospitals within the municipality were randomly sampled. A total of 5 hospitals from Durban and one outlying hospital in nearby Pietermaritzburg with rotating surgical registrars from Durban were included in the study. Therefore a total of 6 provincial public hospitals were included in this study.

### Target population

Medical doctors, professional nurses and patients at selected public hospitals were randomly targeted to participate in this study.

### Inclusion criteria

Almost all medical doctors, professional nurses and patients within the selected hospitals were eligible to participate in the study.

### Sample size

Preliminary sample size for each group of study participants was calculated using a web based freely accessible sample size calculator, Raosoft^® ^[47]. Based on the formula for sample size and margin of error from Raosoft, the estimated sample size for each category of participants was for the recruitment of 360 medical practitioners; 373 professional nurses and 385 patients. Giving a total estimated sample size of 1118 participants. Available data on healthcare personnel indicated that that there were about 5670 medical doctors and 24360 professional nurses registered in KZN in 2010, although there are disagreements on the total number of doctors practicing within South Africa and its provinces with high mobility and vacancy rates [33,48]. Due to the fact that hospitals within the municipality serve as institutions for training of doctors and nurses, there is a constant rotation of medical personnel throughout the district and the province, and since the results were to be extrapolated to the practice of doctors generally in South Africa, I based my initial estimates on the total number of doctors and nurses practicing within KZN province as obtained from health personnel statistics [49]. Overall, because of the low numbers of health personnel, there was minimal difference in estimated sample size regardless of whether sample calculations were based on healthcare professionals within the municipality, practicing in the public sector, or within the province [47,49].

### Data analysis and statistical methods

Data from the questionnaires were captured directly into statistical package for social sciences (SPSS) by a research assistant. The captured data was then checked for completeness and accuracy by the PI (SCC) and a qualified biostatistician. Data was later analyzed using SPSS (version 21) [50]. Descriptive statistics such as proportions, median, mode and interquartile range were used to summarize the data. Scores for comprehension, understanding, information disclosure, voluntariness and informed consent aggregate scores were worked out from the responses. The Mann-Whitney test was used to examine the difference in scores between different categories of healthcare professionals in public hospitals. Kruskal-Wallis test was used to examine the relationship between area of specialization and scores, and occupational rank and scores. Chi-square or Fisher's exact tests were used to test for association between categorical variables in the study.

### Ethical considerations

Ethical approval was obtained from a sub-committee of University of South Africa (UNISA) Research Ethics Committee. The study including the biostatistics methodology was also reviewed and approved by the health research and knowledge management sub-component of the KZN Department of Health. Approval was also obtained from the CEOs or medical managers of each of the randomly selected hospitals included in the study. Finally, written informed consent was obtained from each participant after full information disclosure prior to participation in the study.

## Results

**Figure 1:**
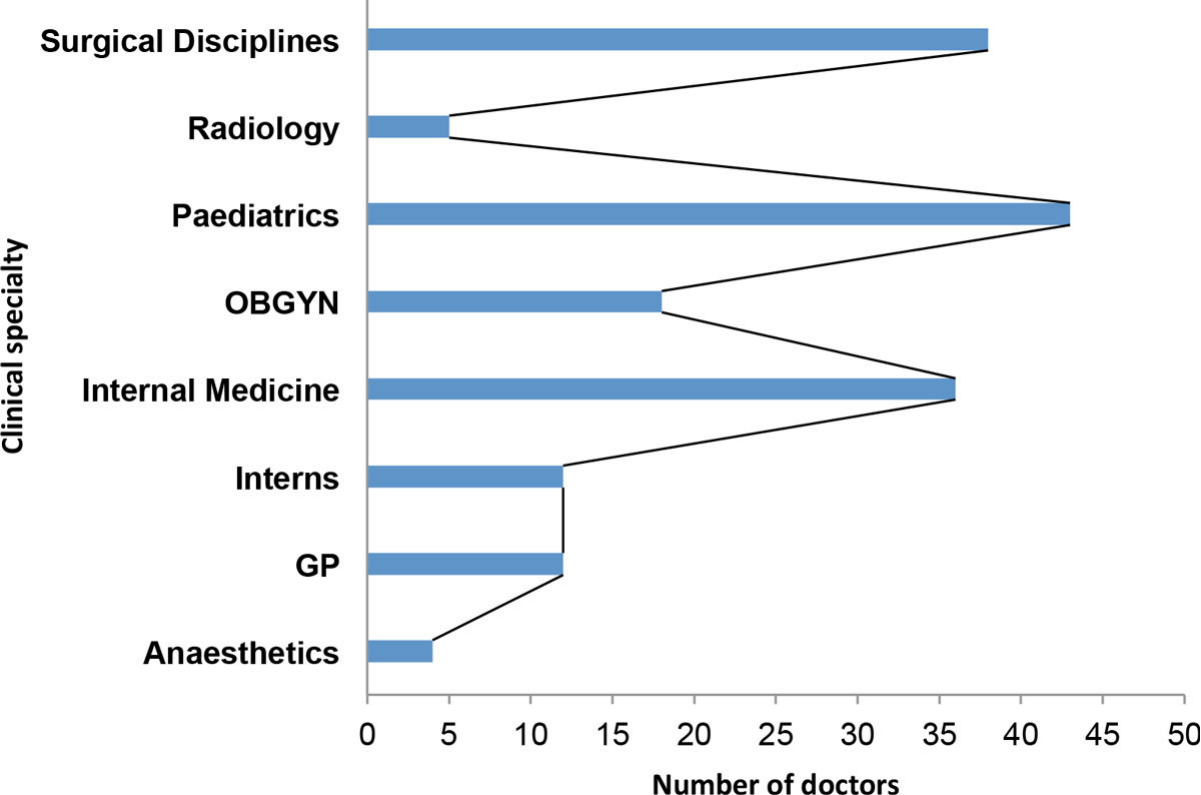
**Participating doctors by clinical sub-discipline or specialty**.

**Figure 2:**
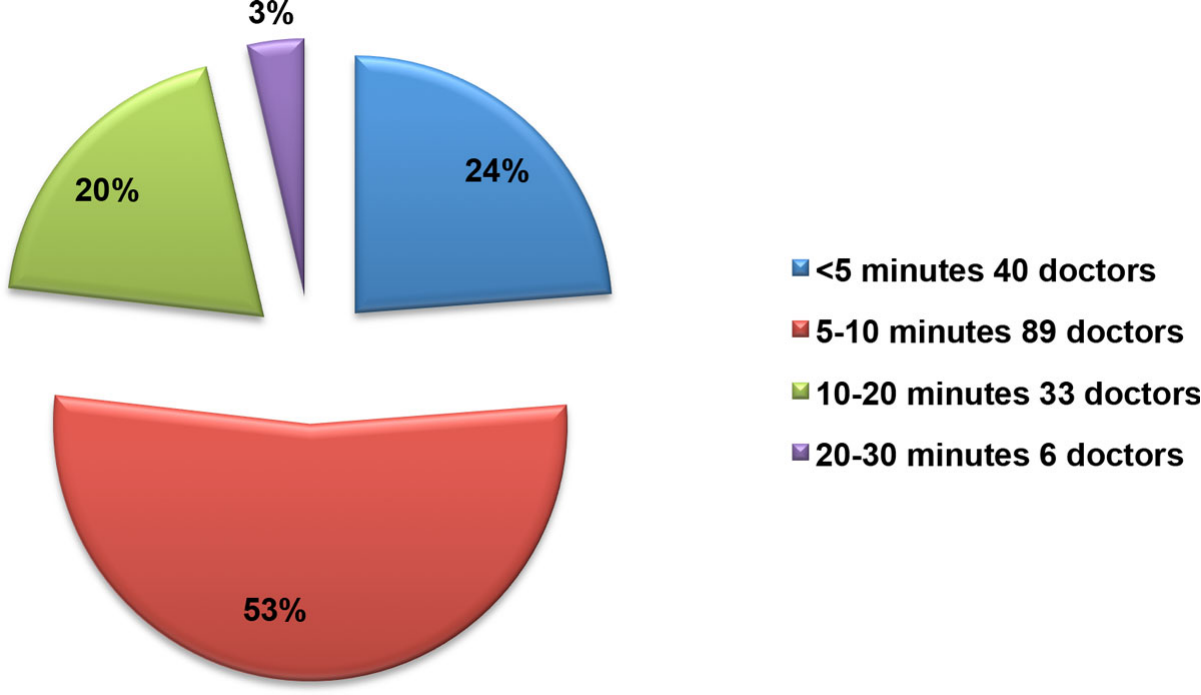
**Time spent by doctors on giving information to patients**.

Demographic characteristics of participating doctors are as shown in Table 1. There was a broad representation of all clinical specialties with participating doctors from all major clinical specialties (Table 1). The overall response rate for this study was 85%, with a total of 946 respondents including doctors, nurses and patients, out of an initial estimate of 1118 participants. After a critical review of captured data a total of 19 participants were excluded due to ineligibility. Therefore a total of 927 individuals were finally included in the study, comprising 168 doctors, 355 professional nurses and 404 patients. Here I report the results of doctor's responses to the questionnaires on the quality of informed consent. The response rate for doctors was 47% of initial estimates. The cohort of participating doctors was then regrouped into 8 major clinical disciplines or specialities for further analysis (Figure 1). The average number of patients seen by doctors in this cohort ranged from 1 to 120 patients/day (median = 20 patients/day). The majority of doctors spent about 5 to 10 minutes providing information to patients prior to treatment decision (Figure 2). When asked whether the amount of time was sufficient, 55.4% of doctors answered 'yes' (Table 2). Those who thought the time spent was inadequate gave various reasons including language barriers and uneducated patients requiring more time for explanations. Others complained of time constraints, administrative responsibilities and large patient numbers being factors militating against spending more time explaining procedures in order to obtain valid informed consent from patients (Table 3). While others explained that the time spent depends on the procedure. Some stated that the time spent was "definitely inadequate" with comments like "in an ideal world, patients [should be] counselled for at least 30 minutes with enough time for questions and clarifications".

**Table 1:** Participant demographics

Doctor characteristics		Valid percent (%)
***AGE***		
Median	30 years	
Range	22-77 years	
***Gender ***		
Male	78	49.1
Female	81	50.9
Missing data	9	-
***Occupational Ranks***		
Interns	47	28
Registrars	44	26.2
Medical Officers (MO)	26	15.5
Consultant/Specialists	51	30.4
Total	168	100

***Clinical disciplines/ sub-disciplines***		
Paediatrics	42	25
Obstetrics and Gynaecology	18	10.7
Internal Medicine	23	13.7
General Surgery	13	7.7
Urology	11	6.5
General Practice (GP)	11	6.5
Orthopaedic s	8	4.8
Dermatology	5	3
Radiology	5	3
Anaesthetics	4	2.4
Cardiology	2	1.2
Gastroenterology	2	1.2
HIV Medicine	1	0.6
Emergency Medicine	1	0.6
Maxillofacial Surgery	1	0.6
Neurology	1	0.6
Neonatology	1	0.6
Oncology	1	0.6
Medical management	1	0.6
***Practice location***		
Public	166	99.4
Private	1	0.6
Missing data	1	-

**Table 2:** Information given to patients by doctors prior to obtaining consent

Information disclosed to patients	Yes (%)	No (%)	Don't know (%)
Diagnosis	162 (96.4)	6 (3.6)	-
Treatment options	136 (81)	32 (19)	-
Recommended treatment	149 (88.7)	19 (11.3)	-
Risk of refusing recommended treatment	140 (88.3)	28 (16.7)	
Cost of medical treatment	20 (11.9)	148 (88.1)	
Information on general risks	147 (87.5)	21 (12.5)	
Information on benefits	150 (89.3)	18 (10.7)	
Information on right of refusal	109 (64.9)	59 (35.1)	
			
***Probing questions***			
Do you think the information you provide is sufficient?	121 (72)	27 (16.1)	19 (11.4)
Do you think this amount of time spent is sufficient?	93 (55.4)	66 (39.3)	9 (5.4)
Do you think the hospital consent form is adequate?	105 (62.5)	51 (30.4)	12 (7.1)

**Table 3:** Major challenges to obtaining informed consent by doctors

Challenges	Median score	P-value
Lack of admin. support e.g. interpreters	4	0.013
Time constraints	2	0.226
Work load	3	0.110
Lack of education	4	0.915
Cultural barriers	5	0.551
Language barriers	2	0.453
Medical paternalism (doctor knows best)	7	0.300

Notes: (a) Challenges were ranked from 1-7, with 1 being most difficult and 7 being least difficult, median scores are reported here. (b) Tests of statistical significance across all clinical disciplines were done using Kruskal-Wallis test for independent samples, significance level is P = 0.05

### Information given to patients before obtaining consent

When asked about what types of information was generally disclosed to patients prior to obtaining consent. The majority of doctors provided information on '*diagnosis*' (96.4%), 89.3% provided information on the '*benefits of treatment*', 81% provided information on '*treatment options'*, 88.7% recommended a specific treatment. About 83.3% gave information on '*risk of refusing treatment'*, while 64.9% advised patients on *'the right of refusal*'. Only 11.9% of doctors provided information on the '*cost of treatment' *(Table 2). When asked specifically whether they explained the benefits of the procedure to a patient, 97% of doctors answered affirmatively, while 95% explained the risk of the procedure to patients (Table 4). When doctors were asked whether they thought the amount of information provided to patients was sufficient for valid informed consent, 72.5% answered 'yes', 16.1% answered 'no'; while 11.4% answered 'don't know'.

**Table 4:** Nature of risks disclosed to patients

Types of risks disclosed	Yes (%)	No (%)	Don't know (%)
Most serious risks	144 (85.7)	18 (10.7)	2 (1.2)
Most common risks	152 (92.1)	13 (7.9)	-
All material risks	35 (21.2)	117 (70.9)	13 (7.9)
Do you explain risks of the procedure to patients?	158 (94.6)	8 (4.8)	1 (0.6)
Do you explain benefits of the procedure to patients?	162 (97)	4 (2.4)	1 (0.6)

### Hospital consent form

When asked whether the current consent form used to obtain informed consent from patients is adequate, 62.5% (105) doctors thought it was adequate, while 30.4% (51) answered 'no' and 7.1% (12) answered 'don't know'. When asked to explain why the current universal consent forms used in public hospitals was inadequate, many doctors complained that the current form does not give opportunity to detail specific complications because different clinical conditions may require different mandatory disclosures. Some suggested that the consent forms should contain tick-boxes for more detailed information disclosure. Others complained that the current form does not take into account "privacy, language and cultural values". For example the form is "done briefly in a language not the patients first language (sometimes cannot get an interpreter), so we take for granted the patients understands when he/she says yes to everything". Others complained that the form contains "no binding space that consent was given or alternatives discussed" or that it was "not specific to minors; when guardian details must be recorded". Others concluded that the current form has not "not kept up with current progress in medico-legal teaching".

### Nature of risks disclosed to patients

Information about specific risks of each procedure was provided to patients by about 95% of doctors. When asked what *types of risks *were disclosed to patients? About 92% of doctors said they disclosed the *'most common risks'*, 86% disclosed '*the most serious risks'*, while only 21% disclosed '*all material risks' *to patients (Table 4). Chi-squared tests were used to test for statistical significance on the types and nature of information disclosed to patients across different clinical specialties. Information on disclosure of 'clinical diagnosis' was statistically significant (p ≤ 0.001), with radiologists least likely to give patients information on diagnosis. Similarly there was statistical difference in disclosure of information on 'recommended treatment' (p = 0.002), with anaesthetists and radiologists least likely to recommend treatment to patients. Finally information disclosure on 'treatment options" was also statistically significant across different specialities (p = 0.004), with 60% of radiologists, 50% of anaesthetists and 32.6% of paediatricians least likely to discuss treatment options with their patients. All other categories of information disclosed were not statistically significant across different clinical specialties (Table 2).

### Methods used to obtain consent from patients

When asked how patients normally provide consent for clinical procedures. About 6.7% of doctors said 'verbally', 50.9% answered 'written', 34.5% said both verbally and written, while 7.9% answered 'it depends'. Doctors who answered 'it depends' gave various reasons for obtaining consent using different formats. Most stated that it depends on the type of procedure. Others said it depends if it is an 'emergency' or if the patient is unconscious or a minor. Others obtained telephonic consent when parent/guardian was not available, while others said sometimes the hospital superintendent would give the necessary consent in an emergency. Some doctors said it depends if written consent is required by law. There was no statistical difference across specialities or occupational ranks in methods of obtaining consent (p = 0.587).

### Comprehension/understanding of information disclosed

To examine the extent of patients understanding of informed disclosed by doctors, we asked questions about the language and methods used to obtain informed consent from patients. When communicating with patients, 64.3% (108) doctors used '*English language'*, 44.6% (75) used the '*patients' local language*', while 69% (116) doctors said they used '*both English and the patients local language*'. To enhance or facilitate understanding of information disclosed to patients, 96.4% (162) doctors used '*words*' or communicated verbally, 20.2% (34) used '*pictures*', 41.7% (70) used '*diagrams*', while 72% (121) used '*interpreters*' to communicate with patients. When doctors were asked if they think patients understood the information given to them; 76.4% (126) answered 'yes'; 3.6% (6) answered 'no'; 12.7% (21) answered 'don't know', while 7.3% (12) said they 'didn't think so'.

### Competence or capacity to give informed consent

When asked whether they generally presumed that patients had the capacity to consent to treatment, 67.3% (113) doctors answered 'yes', 31% (52) answered 'no', while 1.8% (3) answered 'don't know'. When asked whether they routinely assessed a patient's capacity to give consent to treatment, 58.9% (99) doctors answered 'yes', 37.5% (63) answered 'no'; while 3.6% (6) said they 'don't know'. When asked to rank the most important factors in assessing patients' capacity, 73% (123) doctors ranked '*level of consciousness*' first, 74% (125) ranked '*age*' second; 72.6% (122) ranked '*educational level*' third, 65.5% (110) ranked '*appearance*, fourth while 66.67% (112) ranked '*sex*' of the patient last in terms of importance.

### Methods used to assess capacity

When asked to rank methods used in assessing patients' capacity when confronted with difficult cases, 72.6% (122) doctors ranked '*mental status examination*' first, 70.8% (119) ranked '*psychiatric consultation*' second, 66.1% (111) and 58.9% (99) doctors ranked '*ethics consultation' *and '*use of surrogates*' equally third respectively, while '*court adjudication*' was ranked fourth by 62.5% (105) doctors. About 28.6% (48) of doctors said they would use '*none of the above*' methods. When asked to specify what method they routinely used in assessing patients capacity when dealing with difficult cases, majority of doctors said they used a mini- mental status exam (MMSE), followed by level of consciousness or orientation in time place and person, and the Glasgow coma scale (GCS) in difficult cases. Others said they would involve parents/guardians especially in paediatric cases, while some said they would use other surrogates such as a social worker/psychologist, family members or the hospital superintendent. There was no significant difference across clinical specialties in terms of 'presumption of capacity' (p = 0. 110) or routine assessment of capacity (p = 0.698).

### Consent in emergency situations

When doctors were asked whether they obtained consent in emergency cases, 54.2% (90) doctors answered 'yes', 19.9% (33) answered 'no', 24.1% (40), said 'it depends', while 1.8% (3) said they 'don't know'. Doctors who answered 'it depends' gave various reasons for not obtaining consent in emergency cases, including level of consciousness or mental status of the patient, availability of parent or guardian to serve as surrogate. Others said if the patient was in a stable condition and able to comprehend, then they would obtain consent. Others said if patient is incapacitated, then proxy consent is obtained from the consultant or medical superintendent of the hospital. Others indicated that it depends on the procedure and whether it was a life threatening situation.

### Voluntariness and consent to treatment

When doctors were asked whether they would 'allow patients to choose a medical procedure or treatment', 53% (88) doctors answered 'yes', 44.6% (74) said 'no', while 2.4% (4) answered 'don't know'. To further explore whether doctors allowed their patients to exercise choice or act on their own free will during clinical encounters, doctors were asked about their understanding and use of implied and presumed consent in practice.

### Implied or presumed consent practices

**Figure 3:**
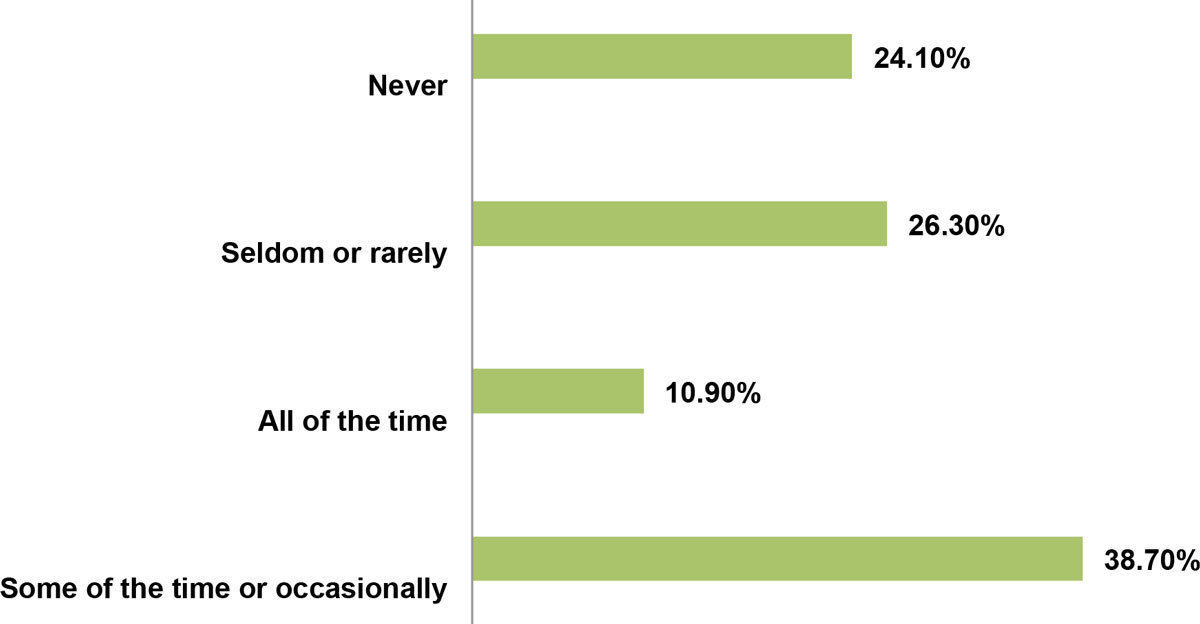
**Use of implied or presumed consent by doctors**.

When doctors were asked whether they ever used implied or presumed consent when treating patients, 53% of doctors said 'yes', while 47% answered 'no'. More doctors said they used implied consent in an emergency rather than in the than in the hospital wards or clinics (Table 5). When asked how often they used implied or presumed consent in practice, about 39% of doctors used implied or presumed consent sometimes or occasionally, while 26% used it on rare occasions. Only about11% used it all of the time, while 24% said they never used it at all (Figure 3). About 66% of doctors also said they obtained specific consent for certain procedures, especially for minor and major surgical procedures or blood transfusions (Table 5). The issues surrounding voluntariness and consent to treatment will be evaluated further from the point of view of patients, when patients' data are analysed.

**Table 5:** Use of implied or presumed consent in clinical practice

Implied/presumed consent	Yes (%)	No (%)	Don't know (%)
Do you ever use implied/presumed consent in practice?	80/168 (53)	71/168(47%)	
When do you use implied/presumed consent:			
1. When patients' present at the clinic?	49/168 (34)	95/168 (66)	1/168 (1)
2. When patients are admitted to the ward?	45/168 (31)	98/168 (68)	1/168 (1))
3. In an emergency?	69/168 (48)	73/168 (50)	3/168 (2

How often do you use implied/presumed consent?			
Some of the time or occasionally	53/168 (38.7)		
Seldom or rarely	36/168(26.3)		
All of the time	15/168 (10.9)		
Never	33/168 (24.1)		
Do you obtain consent for other specific procedures?	95/168 (66)	49/168 (34)	

### Major challenges to obtaining informed consent

**Figure 4:**
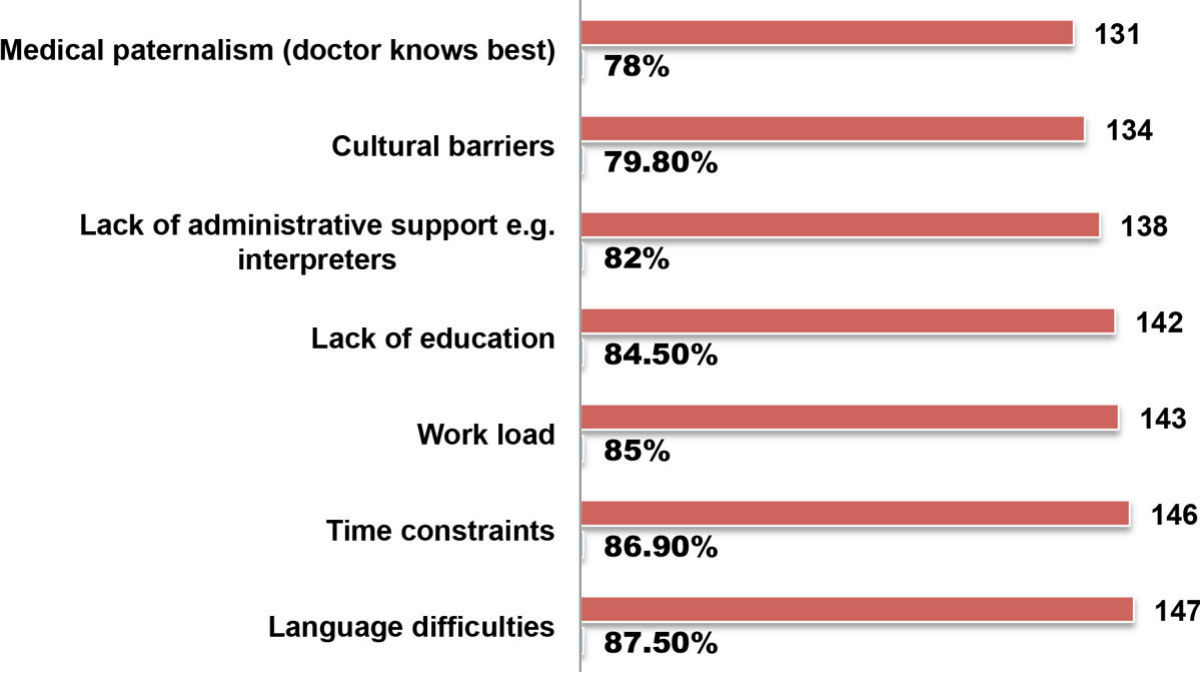
**Challenges to obtaining informed consent by doctors**.

Doctors were asked to rank a series of potential challenges experienced while obtaining informed consent in practice, on a seven point scale of 1-7, with 1 being most difficult and 7 as least difficult (Table 3). The major challenges identified by doctors in this setting included *'language difficulties'*, ranked highest by 87.5% of doctors, '*time constraints' *ranked second by 86.9% doctors, followed by '*work load' *85%, *lack of education *84.5%, and *lack of administrative support e.g. interpreters' *82% of doctors. The least important constraints identified were *'cultural barriers'*, by 79.8%, while *medical paternalism (doctor knows best)' *was ranked last by 78% of doctors (Figure 4). Cultural barriers identified by doctors included religious beliefs such as Jehovah's witnesses or cultural abhorrence of organ transplantation, amputations and blood transfusions. The need to obtain approval from husbands or family members prior to giving consent, preference for traditional healers, cultural taboos, and 'disempowered caregivers' according to one respondent. A test of statistical significance using the Kruskal-Wallis test for independent variables, showed that the 'lack *of administrative support e.g. interpreters' *was statistically significant across all clinical specialities (p = 0.013) (Table 3).

### General knowledge of basic informed consent laws and regulations

To test for general know of informed consent laws and regulations in South Africa, doctors were asked some specific questions. When asked to select the current age of consent to routine treatment in South Africa, only 70.7% of doctors were able to correctly answer '12 years'. This question was answered wrongly by many doctors with 10.8% saying '15 years', 15.3% answered '18 years'; 1.9% answered '21 years', while 1.3% did not know. Further, when asked to select the correct age when women can consent to termination of pregnancy (TOP) in accordance with South African law, only 29.6% of doctors correctly answered 'any age'. Majority gave the wrong answer with 50.9% choosing '12 years', 13.2% chose '15 years', 3.8% chose '18 years', while about 2.5% did not know the correct age. Chi-squared tests were used to test for statistical significance in terms of general knowledge of informed consent laws and regulations across all specialities. There was no statistical significance detected in terms of age of consent, age for women to request for TOP, or standards of information disclosure.

### Responsibility for obtaining consent

When asked whose responsibility it was to assure that adequate information was provided before informed consent, only 61.7% (100) doctors thought it was the *'doctor or healthcare professional's responsibility'*. About, 41% (66) answered that *'both the doctor and patient were jointly responsible'*, while 5% (8) thought it was '*the patient's responsibility'*.

### Standards for information disclosure

When asked whether the current standards for information disclosure were based on a 'reasonable doctor' or 'prudent patient standard'. Most doctors, 60.2% (97) answered that was based on a *'reasonable doctor standard'*, while 47.8% (76) correctly answered *'prudent patient standard'*. When asked whose duty it was to obtain consent from patients in practice, 66.3% (110) doctors correctly answered that it was responsibility of the '*doctor performing the procedure or treating the patient'*. About 6.1% (10) doctors said *'nurses'*, were responsible, 44.6% (74) said *'junior doctors' *were responsible, while 10.8% (18) thought it was the responsibility of *'any available healthcare professional'*, 3.6% (6) doctors did not know.

### Informed consent aggregate scores (ICAS)

**Figure 5:**
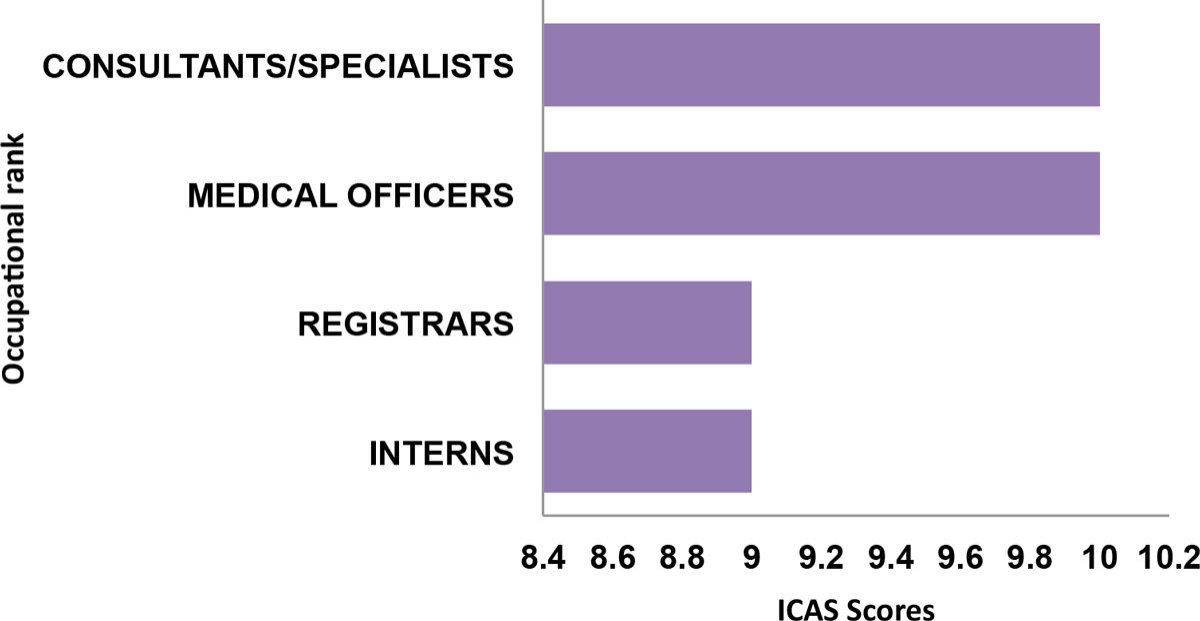
**ICAS of doctors by occupational rank**.

**Figure 6:**
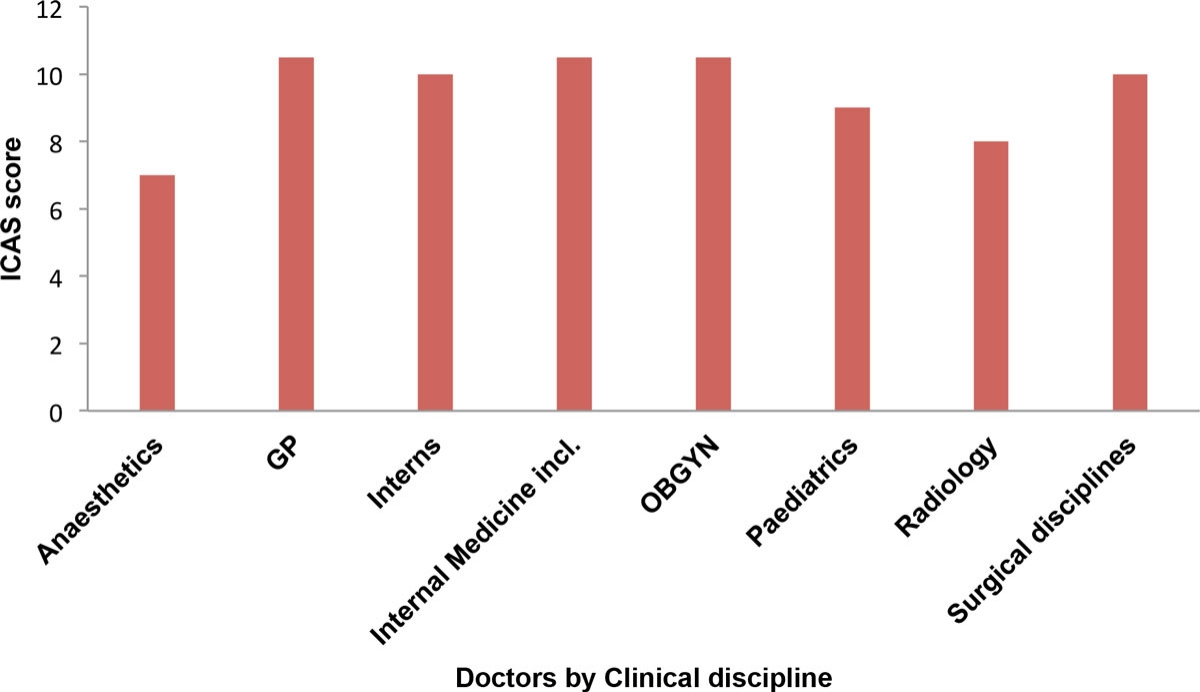
**ICAS scores of doctors by clinical sub-discipline or specialty**.

To compare informed consent practices across occupational ranks of doctors and nurses, as well as between clinical specialties. I developed an aggregate score using a modified version of the method described by Sugarman and others [51]. While the previous authors used a series of seven questions derived from a brief informed consent evaluation protocol (BICEP) during research studies [51]. Here I have selected a series of questions from the questionnaire which relate to information disclosure, voluntariness, assessment of capacity and understanding or comprehension (Table 6). A total of twelve questions from the questionnaire were adjudged to satisfy these criteria. Each of the selected questions was given a rank score of one (1) and the aggregate score is the sum of the scores (12) (Table 6). ICAS aggregate scores for all doctors by occupational rank ranged from 1 to 12, with a median score of 10 (SD = 2.28). The lowest scores were recorded by interns and registrars with a median score of 9, while medical officers and consultants/specialists recorded a median score of 10 respectively (Figure 5). Tests of statistical significance for ICAS scores by occupational rank of doctors was not statistically significant (p = 0.174). However, comparison of ICAS scores by clinical speciality using the Kruskal-Wallis test was statistically significant (p = 0.005). In this case anaesthetists and radiologists had the lowest ICAS scores with a median score of 7 and 8, respectively, while the highest scores were obtained by OBGYN, Internal medicine and GP doctors with a median score of 10.50 (Figure 6). Finally when the ICAS scores of doctors was compared with that of professional nurses. Scores by professional nurses was lower than that of doctors with a median score of 8, while the median score for doctors was 10. The difference in scores between doctors and nurses was highly statistically significant (p ≤ 0.001), using the Mann-Whitney U test for independent samples at a significance level of 0.05.

**Table 6:** Questions used to calculate ICAS

*A. Information disclosure:*	ICAS Score	
What information do you routinely provide to your patients?		
	Yes	No
Diagnosis	1	0
Treatment options	1	0
Recommended treatment	1	0
Risks of refusing recommended treatment	1	0
General risks	1	0
Benefits	1	0
Right of refusal	1	0
*B. Capacity/Competence*		
Do you routinely assess the competence of your patients to consent to treatment?	1	0
Do you generally presume that your patients have the capacity to consent to treatment?	1	0
*C. Voluntariness*		
Do you allow your patients to choose a procedure or particular treatment?	1	0
*D. Understanding*		
Do you think your patients understand the explanations given to them?	1	0
*E. Consent or agreement*		
Do you think the information you provide is sufficient to procure valid informed consent?	1	0
Total: Informed consent aggregate score (ICAS)	12	0

**Note: **The question about cost of medical treatment is excluded from this ICAS calculation in this cohort because the cost of healthcare services public in hospitals is free

## Discussion

Most studies evaluating the quality of informed consent especially in developing countries have focused on informed consent practices in clinical research. These include previous studies from Nigeria [52], Uganda [53], South Africa [54] and Mali [55]. Most of these studies reported problems with comprehension and understanding of the informed consent process by patients including the right of withdrawal [52-55]. Other studies from developed countries have contended with problems of subject's therapeutic misconception, voluntariness and measurement of capacity to consent during biomedical research and clinical trials [51,56-58]. While many studies on informed consent have focused on clinical trials and biomedical research, very few studies have actually looked at the quality of informed consent in clinical practice, especially in Africa [59-61]. The paucity of studies in the area of clinical practice is surprising considering that patients or individuals are more likely to seek treatment for routine medical care than be involved in biomedical research. Nonetheless most of the studies from developing country settings have highlighted the need for more education in biomedical ethics for researchers, healthcare practitioners, as well as patients or human subjects of biomedical research [52-55]. Some studies have identified the need to improve the quality of informed consent documents, including the need for simplified language to enhance participant understanding [62]. Others have highlighted the different notions of informed consent such as the moral and legal dimensions of consent which have the potential to impact on the quality and practice of informed consent, including information disclosure, understanding and shared decision making [63].

### Standards of information disclosure

One of the more controversial areas of informed consent in practice has hovered around the amount of information disclosure required before consent can be considered valid. On this consideration there are two contesting schools of thought. One is the 'reasonable doctor standard' based on English common law as outlined by McNair J in *Bolam v Friern HMC *[64] generally known as the *Bolam test*, which states that: "A doctor is not guilty of negligence if he has acted in accordance with the practice accepted as proper by a responsible body of men skilled in that particular art...". It has been argued that English courts have opted for a paternalistic approach by following the reasonable doctor standard which bases disclosure on the clinical judgement or accepted practice or substantial risk/normal/usual risk principles as established in *Bolam *[22,23] and reaffirmed by the House of Lords in the case of *Sidaway *case where Lord Templeman argued that [65]:

"At the end of the day, the doctor bearing in mind the best interests of the patient and bearing in mind the patients right to information which will enable the patient to make a balanced judgement, must decide what information should be given to the patient, and what terms that information should be couched..."

However, Lord Scarman in the same case suggested the use of a 'prudent patient standard' arguing that: " It was a strange conclusion if our courts should be led to conclude that our law...should permit doctors to determine in what circumstances...a duty arose to warn." [65]. The Courts in North America, have maintained in cases such as *Canterbury v Spence *[17] and *Reibl v Hughes *[66] that a patient must be informed of all material risks, where those 'material risks' would consist of what a reasonable person, in such a patients position, would be likely to attach significance to, in deciding whether to accept or forego a proposed treatment. In South African case law the issue of how much information should be disclosed to patients has been the subject of debate since *Lymberg v Elliot *[67] where the Court was of the opinion that a 'doctor is not obliged to disclose all the conceivable complications that may arise during a medical procedure.' However in *Castell v DeGreef *the Court concluded, that a doctor is obliged to warn the patient of all the 'material risks' inherent in the proposed treatment. Where material risks is based on a 'prudent patient standard' [26]. Therefore the current requirements for information disclosure in South Africa are consistent with the practice in North America as outlined in section 6 of the NHA [28]. These requirements reaffirm the need for disclosure of all material risks with few exceptions. In the current study, the results show that while the majority of South African doctors complied substantially with the requirements of the NHA in terms of information disclosure (Table 2), only about 21% of doctors complied with the 'material risks' standard in terms of risk disclosure (Table 4). Further, a majority of doctors (60%) chose the 'reasonable doctor' rather than the 'prudent patient' standard as the required standard for information disclosure in clinical practice. Therefore the current practice by doctors in terms of information disclosure is inconsistent with ethical guidelines from the HPCSA [68] or current local laws [26-28].

### Comprehension of information disclosed

It has suggested that in developing countries such as South Africa, where education standards and literacy levels are low, knowledge and power asymmetry usually exist between patients and health care professionals [34,35]. It is also important to recognize the historical backdrop of colonialism and racism, and ongoing challenges of poverty and exploitation [30-32]. In spite of such considerations however, doctors still have an obligation to adequately explain clinical procedures to patients without turning them or surrogates into students of medicine [69]. The ability to use written information is important to comprehension and understanding [70], as such barriers to communication arising from illiteracy and language differences may prevent a common understanding of medical procedures, thereby putting patients at risk of providing consent without comprehension [71]. It is therefore important that healthcare providers ensure that patients understand the proposed treatment or procedure prior to providing consent. Some authorities have suggested a verbal or written test to ascertain patient capacity, competence or understanding before considering informed consent valid [58].

### Language

In the current study one of the major barriers towards obtaining valid consent by doctors has been listed as 'language difficulties', ranked highest by 88% of doctors in this cohort. This was supported by complaints about 'lack of education' (85%) and lack of interpreters by 82% of doctors (Figure 4). Therefore it cannot be overemphasized that one of the major barriers to obtaining valid informed consent in this environment is the issue of language. It has been argued that language barriers can have a deleterious effect on healthcare service delivery, leading to such errors as misdiagnosis, failure of preventive therapy or non-adherence to prescribed medication, which could ultimately lead to charges of medical negligence and award of substantial damages against doctors [72]. The issues of language difficulties and the necessity for appropriately trained interpreters, is not limited to developing countries, but is also a barrier to proper healthcare services delivery in developed countries such as the USA or any multicultural/multilingual society [72]. Currently South Africa has 11 official languages; therefore language barriers, especially the absence of adequately trained interpreters to assist healthcare professionals in providing care to patients is a major problem. In another study at a South African district hospital, the authors concluded that language barriers in hospitals create significant problems for healthcare professionals and can impact negatively on patients' rights to confidentiality, informed consent and the quality of healthcare service delivery [73]. Other cultural barriers identified by doctors in this study include different cultural beliefs about blood transfusion and amputations. The impact of family members in decision-making, especially husbands within the traditional African cultural ethos. All of these can serve as barriers to the appropriate practice of informed consent in developing country settings [34,74]. To further improve understanding and comprehension during the informed consent process, the US National Bioethics Advisory Commission (NBAC) has suggested that *community participation *is acceptable, which may include providing written information sheets for discussions with family members and holding community meetings, but cautions that family permission should not replace the requirement for individual informed consent [75,76].

### Capacity

In the common law there is a presumption that any adult person has the capacity to consent or refuse medical treatment unless proven otherwise by acceptable evidence. A lack of capacity cannot be established merely by reference to a person's age, appearance, and intelligence, level of education, or any condition or aspect of behavior, which might lead others to make unjustified assumptions about capacity [2]. According to the Court in *Richmond v. Richmond *[77]:

*Capacity is ultimately a legal not a medical decision... it is for the court to decide the question of capacity, although the court must pay attention to the evidence of experts in the medical profession who can indicate the meaning of symptoms and give some idea of the mental deterioration which takes place in cases of this kind*....

Thorpe J summarized the common law test for capacity in *Re C *[78] where he said that: the patient must be able to (a) comprehend and retain the information (b) believe it (c) weight it in the balance so as to arrive at a choice. The UK *Mental Capacity Act *[79] further simplified this test, which now states that a person is deemed incapable of making a decision and exercising autonomy rights where that person is unable:

a) To understand the information relevant to the decision, b) To retain that information

c) To use or weigh that information as part of the process of making the decision, or

d) To communicate his decision (whether by talking, using sign language or any other means) [2,78,79]. In the current study about 67% of doctors said that they would presume that patients have the capacity to consent to treatment, although this low percentage may have been influenced by the large number of pediatricians within our study cohort, who would normally assume that their patients could not provide consent based on their age. Similarly, only 59% of doctors in this cohort routinely tested their patients for capacity to prior to treatment. On the other hand the majority of doctors accurately ranked factors such as level of consciousness, age, educational level, appearance and sex, in descending order, as being factors used in the assessment of capacity. Also, only 71% of doctors accurately identified 12 years as the age of consent to routine medical treatment in South Africa, while only 30% of doctors correctly identified the age of consent to TOP as 'any age", as stipulated in the *Choice on termination of pregnancy Act *[80]. This evidence suggests inadequate knowledge of current local laws and regulations on informed consent in South Africa. When assessing capacity in difficult cases, majority of doctors responding said they would use a MMSE, GCS or orientation in time place and person, to ascertain patient's capacity to give consent to treatment. This is contrary to previous studies on capacity assessment tools for medical treatment which concluded that both the MMSE and GCS should be viewed as blunt instruments when determining patients' capacity [56]. Perhaps more sensitive capacity assessment tools, such as the MacArthur Competence Assessment Tool--Treatment (MacCAT-T) should be evaluated for use in this setting [56,58].

### Voluntariness and consent or agreement to treatment

Voluntariness of consent has been one of the more difficult areas to assess by empirical methods because of the variations in patients' clinical condition and cultural norms associated with the concept of voluntariness [14,57,63]. In African traditional societies the influence and respect for family, friends and elders is very important in accordance with cultural ethos. Therefore, it is not unusual for individuals to seek the advice of family, friends and relatives before making important decisions related to healthcare [34,59,63]. While these types of interference may be considered undue influence in western cultures, with their history of libertarian autonomy and individual rights. African societies are more accepting of collective decision making, based on a different concept of autonomy derived from Ubuntu or "*sumus, ergo sum*, (we are, therefore I am)" [81]. It is generally recognized that voluntariness in informed consent means that the patients' consent must be given voluntarily, devoid of any undue influence or coercion either by fraudulent misrepresentation or trickery from the physician or family or friends [14,76]. According to Lord Donaldson in *Re T *[82]:

*If...his will was overborne; the refusal will not have represented a true decision. In this context the relationship of the persuader to the patient-for example, spouse, parents or religious adviser-will be important, because some relationships more readily lend themselves to overbearing the patient's independent will than others*.

In our current study I have tried to study voluntariness by asking some indirect questions from doctors such as whether doctors would allow patients to choose a particular procedure or treatment, only 53% answered affirmatively. Similarly when asked whether doctors ever used implied or presumed consent in practice, 53% answered in the affirmative. When further asked how often they used implied or presumed consent in practice, 39% said occasionally, 26% said rarely, while 11% said all of the time. Only 24% of doctors said they 'never' used implied or presumed consent in practice (Figure 3). This suggests some elements of medical paternalism are still prevalent in clinical practice in this environment. It appears that many doctors resort to implied/presumed consent in lieu of obtaining legally valid consent, contrary to ethical guidelines from the HPCSA, which advices doctors not to simply presume that patients have given consent when they lay down on the examination table [68], consistent with the injunction of the Court in *Stoffberg v. Elliot *[24] which stated that:

A man by entering a hospital does not submit himself to such surgical operations as the doctors in attendance upon him might think necessary...he retains his rights of control and disposal of his own body; he still has the right to say what operation he will submit to, and unless consent to an operation is expressly obtained, any operation performed on him without his consent is an unlawful interference with his right of security and control of his own body."

Perhaps this unquestioned practice could be explained by the power asymmetry that exists between doctors and patients or special respect shown to doctors by patients in this environment as described in another study from Nigeria [59]. It may also be associated with the many challenges experienced by doctors practicing in this environment including heavy workload and lack of administrative support (Figure 4).

### Comparative analysis of ICAS scores

Analysis of ICAS scores showed that interns and registrars scored lower than medical officers and consultant/specialists. This could be explained by the fact that interns and registrars are still trainees and it should be expected that their knowledge of informed requirements would be lower than that of their trainers and supervisors. Across clinical subspecialties radiologists and anaesthetists scored lower than internists and surgeons and GPs. This is somewhat consistent with findings from another study in Croatia where anaesthetists scored lower than internists and surgeons on informed consent [83]. The plausible explanation is that because radiologists and anaesthesiologists are ancillary subspecialties, they may not be required to provide information to patients such as diagnosis treatment options etc. and may also depend on primary care physicians to obtain prior informed consent prior to referral for supplementary services [68]. In the case of nurses and doctors, it should be expected that doctors are more knowledgeable about informed consent regulations, because doctors are generally better trained in the areas of medical law and ethics and are required to make final decisions regarding patient care, therefore the requisite knowledge about regulations and practice maybe more rigorously enforced by the regulatory authorities.

## Conclusions

Previous studies on informed consent in Africa have shown that while doctors are generally knowledgeable about the ethical doctrine of informed consent, the application and adherence to the legal and ethical requirements of informed consent is usually lacking in practice. Analysis of data from this study confirm these observations by showing that doctors practicing in public hospitals in South Africa are generally knowledgeable about some aspects of informed consent, such as information disclosure. However not all adhered to the critical elements as specified in the *NHA*, or the requirements based on international standards of care or local ethical guidelines. The major challenges militating against the proper practice of informed consent as identified in this study were related to issues of language barriers and lack of administrative support, especially interpreters to assist with communicating with patients. Others factors identified include large patient numbers with associated time constraints and workload. These results show that while the majority of doctors spent an average of 5-10 minutes on obtaining informed consent, this amount of time was considered inadequate by many doctors. Knowledge of essential local laws such as the age of consent for routine medical treatment or age of consent for TOP in South Africa was not universally known by doctors. Similarly, the majority of doctors still believed in the paternalistic concept of a 'reasonable doctor standard' rather than more currently accepted 'prudent patient standard' and the disclosure of all material risks. This study suggests that doctors were statistically more knowledgeable about informed consent than professional nurses, however it remains to be seen whether this translates into clinical practice. Finally, there was evidence of overuse of implied and presumed consent by doctors with implications for medical paternalism and lack of voluntariness in consent. This study was limited to public hospitals in an urban setting and the study period was restricted to 3 months. It is possible that future studies in private hospitals or in a more rural setting may provide different results. Based on the findings in this study, one can recommended the recruitment and training of a 'corps' of interpreters as part of medical teams in South African hospitals, to assist in improving the quality of doctor-patient communications, informed consent, confidentiality, and healthcare service delivery in public hospitals. It would also be useful to modify the current universal hospital consent form to better reflect current teaching in medico-legal practice, by including translations in local languages, or options for specific consent for certain procedures or mandatory disclosures as required by law. It would also be useful for patient information leaflets to be produced in local languages to enhance patient education and understanding prior to providing consent. Finally, continuing education for doctors and other healthcare professionals in ethics and medical law will go a long way towards improving the overall quality of healthcare service delivery in South African hospitals.

## List of Abbreviations used

BICEP: brief informed consent evaluation protocol; CEO: Chief executive officer; GCS: Glasgow coma scale; GP: general practitioner; HCW: healthcare workers; HPCSA: Health Professions Council of South Africa; IBC-international bioethics committee; IBM: international business machines; ICAS: informed consent aggregate score; KZN-KwaZulu-Natal; MMSE: mini mental status examination; MacCAT-T: Macarthur competence assessment tool: treatment; MO-Medical Officer; NBAC: National bioethics advisory Commission; NHA: national Health act; NHS: National Health Service; OBYGYN-obstetrics and gynecology; TOP: termination of pregnancy; SD: standard deviation; SPSS: statistical packages for the social sciences; UK: United Kingdom; USA: United States of America, US: United States, UDHR: Universal declaration of human rights, UKZN: University of KwaZulu-Natal; UNESCO: United Nations Educational Scientific and Cultural organization; UNISA-University of South Africa.

## Competing interests

This paper is partly derived from a research project entitled "An investigation of informed consent in clinical practice in South Africa", which is being completed as a thesis for the award of the LLD (Doctor of Laws) degree at the University of South Africa (UNISA).

## Authors' contributions

Author conducted the research study and wrote the manuscript

## Supplementary Material

Additional file 1 **Questionnaire for healthcare professionals (doctors and professional nurses)-Final**. Click here for file

## Declarations

Publication of this supplement has been funded by the College of Health Sciences and the Research Office at the University of Kwazulu-Natal.

This article has been published as part of *BMC Medical Ethics *Volume 14 Supplement 1, 2013: Selected papers from the 3rd Ethics, Human Rights and Medical Law Conference (3rd EHMRL). The full contents of the supplement are available online at http://www.biomedcentral.com/bmcmedethics/supplements/14/S1.
