# Supplementary material for: Evaluating the quality of informed consent and contemporary clinical practices by medical doctors in South Africa: An empirical study
Source: BMC Med Ethics. 2013 Dec 19;14(Suppl 1):S3. doi: 10.1186/1472-6939-14-S1-S3 (PMC3878312; doi:10.1186/1472-6939-14-S1-S3)
Supplement: Additional file 1 — Questionnaire for healthcare professionals (doctors and professional nurses)-Final. [file 1472-6939-14-S1-S3-S1.docx]

**Questionnaire for Healthcare professionals (doctors and professional nurses)**

SECTION A

DEMOGRAPHICS

1. Age of the Respondent:

2. Gender

( ) Male ( ) Female

3. Are you a doctor or a professional nurse? _________________________

4. Years of professional experience or rank________________________

5. Area of specialization, please state ________________________________

6. Department in the hospital ________________________________

7. Public ( ) or Private Practice ( )__________________________

SECTION B

8. How many patients do you see on average in a day? _______________________

9. How much time do you spend giving information about a treatment or procedure in to a patient during a professional encounter?

( ) < 5 minutes ( ) 5-10 mins

( ) 10-20 mins ( ) 20-30 mins

( ) > 30 mins ( ) None

10. Do you think this amount of time is sufficient?

( ) Yes ( ) No ( ) Don’t know

11. If No, Please explain why? ___________________________________________

_________________________________________________________________

12. Do you think the information you provide is sufficient to procure valid informed consent?

( ) Yes ( ) No ( ) Don’t know

13. Do you think the consent form currently used in your hospital is adequate to obtain valid informed consent from patients?

( ) Yes ( ) No ( ) Don’t know

If No, please explain why ____________________________________________

14. What information do you routinely provide to your patients? Please tick or circle all that apply

( ) Diagnosis Y or N ( ) Risks Y or N

( ) Treatment Options Y or N ( ) Benefits Y or N

( ) Recommended Treatment Y or N ( ) Right of refusal Y or N

( ) Risks of refusing recommended treatment Y or N

( ) Costs of medical treatment or each option Y or N

Any additional information? (please specify)____________________________________

________________________________________________________________

15. Do you allow your patients to choose a procedure or particular treatment?

( ) Yes ( ) No ( ) Don’t know

16. Do you explain the benefits of the procedure to the patient?

( ) Yes ( ) No ( ) Don’t know

17. Do you explain the risks of the procedure to the patient?

( ) Yes ( ) No ( ) Don’t know

18. If yes, what types of risks do you routinely explain to the patient?

A. Most common risks ( ) Yes ( ) No ( ) Don’t Know

B. Most serious risks ( ) Yes ( ) No ( ) Don’t Know

C. All material risks ( ) Yes ( ) No ( ) Don’t Know

19. What language do you use to explain/obtain informed consent from your patients?

A. English ( ) Yes ( ) No ( ) Don’t know

B. The patients local language ( ) Yes ( ) No ( ) Don’t know

C. Both English and local language ( ) Yes ( ) No ( ) Don’t know

20. Which of the following methods do you use to explain/obtain consent from patients? Please tick all that apply.

( ) Words ( ) Diagrams

( ) Pictures ( ) Interpreter

( ) None

21. Do you think your patients understand the explanations given to them?

( ) Yes ( ) No ( ) Don’t know ( )Don’t think so

22. Do you routinely obtain consent in emergency cases?

( ) Yes ( ) No ( ) Don’t know ( ) It depends

If you choose it depends, please explain_______________________________

_______________________________________________________________

23. How do the patients normally provide consent?

( ) Verbally ( ) Written ( ) Both ( ) It depends

If you choose it depends, please explain_______________________________

_______________________________________________________________

24. Who obtains informed consent from patients in your practice or clinic

A. Nurses

B. Junior doctors

C. The doctor performing the procedure/treating the patient

D. Any available healthcare professional

E. Don’t know

25. What are the challenges you face in the process of obtaining informed consent from a patients in clinical practice?

Please rank in order of importance (where 1 is most important and 7 is least important):

A. Time constraints ( )

B. Work load ( )

C. Language difficulties ( )

D. Lack of administrative support e. g. interpreters ( )

E. Cultural barriers ( ). Please specify__________________________

F. Lack of education ( )

G. Medical paternalism (Doctor knows best) ( )

26. Do you routinely assess the competence of your patients to consent to treatment

( ) Yes ( ) No ( ) Don’t know

27. If Yes please rank the following criteria in terms of importance in assessing patient capacity or competence to consent to treatment (where 1 is most important and 5 is least important):

A. Age ( )

B. Sex ( )

C. Appearance ( )

D. Educational level ( )

E. Level of consciousness ( )

28. Do you generally presume that your patients have the capacity to consent to medical treatment?

( ) Yes ( ) No ( ) Don’t Know

29. In difficult cases, which of the following methods do you/ would you use determine if a patient has the capacity to consent to treatment (Please rank in order of importance where 1 is most important and 6 is least important)

A. Mental Status Exam ( ) Please specify which one_____________________

B. Psychiatric consultation ( )

C. Ethics consultation ( )

D. Court adjudication ( )

E. Surrogates ( ) Please specify_____________________________

F. None of the above ( )

Section C- Generic questions on informed consent

30. Do you have any suggestions or recommendations regarding informed consent?

________________________________________________________________________________________________________________________________________________________________________________________________________________________________________________________________________________________________

31. At what age can a minor consent to routine medical treatment in South Africa? (Please choose one)

12 years ( ) 15 years ( ) 18 years ( ) 21 years ( ) Don’t know ( )

32. At what age can a woman request for termination of pregnancy in South Africa? (Please choose one)

12 years ( ) 15 years ( ) 18 years ( ) Any age ( ) Don’t know ( )

33. In your opinion, which standard do you think should be used for information disclosure before obtaining consent from patients?

A. Based on a reasonable doctor standard ( ) Yes ( ) No ( ) Don’t know

B. Based a reasonable/prudent patient standard ( ) Yes ( ) No ( ) Don’t know

34. Whose responsibility is it to ensure adequate information disclosure before informed consent?

A. ( ) Doctor or healthcare professionals responsibility

B. ( ) The patients responsibility

C. ( ) The patient and healthcare professional are jointly responsible

**Section D:**

35. Do you ever use implied or presumed consent when treating patients? ( ) Yes or ( )No

If yes, when do you usually use implied or presumed consent?

A. When the patient present themselves at the Clinic ( ) Yes ( ) No ( ) I don’t know

B. When the patient is admitted to the Ward ( ) Yes ( ) No ( ) I don’t know

C. In an emergency ( ) Yes ( ) No ( ) I don’t know

36. What do understand by the term implied or presumed consent?

Implied consent: _________________________________________________________

_______________________________________________________________________

Presumed consent: _______________________________________________________

_______________________________________________________________________

37. How often do you use implied or presumed consent when treating patients?

1. Some of the time or Occasionally ( )
2. All of the time ( )
3. Seldom or Rarely ( )
4. Never ( )

38. Do obtain any additional or specific consent for certain procedures? ( ) Yes ( ) No

If yes, please list all or any procedures for which you would obtain specific consent from the patient:

______________________________________________________________________________

______________________________________________________________________________

**Thank you for your assistance**
